# Facile Fabrication of Energetic Nanocomposite Materials by Polydopamine

**DOI:** 10.3390/ijms242216199

**Published:** 2023-11-11

**Authors:** Zhanxin Song, Wei Liu, Mo Xian, Miaomiao Jin

**Affiliations:** 1 Chinese Academy of Sciences Key Laboratory of Biobased Materials, Qingdao Institute of Bioenergy and Bioprocess Technology, Chinese Academy of Sciences, Qingdao 266101, China; songzx@qibebt.ac.cn (Z.S.); wliu@icost.ac.cn (W.L.); 2 Shandong Energy lnstitute, Qingdao 266101, China; 3 Qingdao New Energy Shandong Laboratory, Qingdao 266001, China

**Keywords:** polydopamine, Al/CuO nanothermite, thermal analysis

## Abstract

Polydopamine-based materials have been widely investigated for incorporation in energetic nanocomposites due to their outstanding adherence. However, these materials are often prepared in alkaline environments, which negatively affects Al nanoparticles. In this study, a one-pot assembly was devised for the preparation of a polydopamine-based Al/CuO energetic nanocomposite material (Al/PDA/CuO) in a neutral environment. The CuO and Al nanoparticles of the Al/PDA/CuO nanothermite were uniformly dispersed and closely combined. Consequently, the Al/PDA/CuO nanothermite was able to release more heat (2069.7 J/g) than physically mixed Al/CuO (1438.9 J/g). Furthermore, the universality of using polydopamine in the assembly of different types of energetic nanocomposite materials was verified, including an organic energetic material-nanothermit (HMX/PDA/Al/CuO nanothermite) and an inorganic oxidant-metal nanocatalyst (AP/PDA/Fe_2_O_3_). This study provides a promising route for the preparation of polydopamine-based energetic nanocomposites in neutral aqueous solutions.

## 1. Introduction

Nanothermites are comprised of inorganic oxidizers (iron, copper, bismuth, and other oxides) and metallic fuels (magnesium, aluminum, etc.) [1,2,3,4], and they are widely used in pyrotechnics, airbag ignition materials, and composite solid propellants [5,6,7]. The inorganic oxidizers and metallic fuels can be combined at the nanoscale, and the ratio of oxidizer to fuel should be adjusted to prepare nanothermites with the best heat release properties. The energy release process and release rate of energy are controlled by the mass and thermal transport between the fuel and oxidizer [8,9]. In other words, the inorganic oxidizers and metallic fuels in nanothermites should be tightly combined and uniformly dispersed to achieve an enhanced rate of energy release. However, nanoparticles tend to aggregate, which has limited the application of nanothermites. Various techniques such as sol-gel synthesis, arrested reactive milling, electrostatic assembly, electrophoretic deposition, and self-assembly have been exploited to improve the heat release properties of nanothermites [8,9,10,11,12,13,14]. However, these strategies have several drawbacks, including the use of organic solvents and harsh preparation conditions. Thus, developing an approach for the preparation of energetic nanocomposites under mild conditions would be highly desirable.

Marine mussels can firmly attach themselves to various material surfaces under natural conditions [15]. The proteins in the adhesive foot of a mussel contain chemical moieties such as catechols and amines. Based on the principle of bionics, dopamine (DA) has been reported as being capable of mimicking the adhesive properties of mussel proteins. Dopamine can self-polymerize to form a strong adhesive polydopamine (PDA) film on the surface of various materials [16,17]. PDA can be prepared under mild polymerization conditions, is hydrophobic, and can be easily modified by secondary reactions [18,19]. These unique properties mean that the use of PDA for the surface modification of energetic materials has become a research hotspot.

PDA has been reported to adhere to the surface of Al and exhibits strong chelation with CuO through its catechol unit. Therefore, in this work, PDA was selected as the bonding layer to form an Al/PDA/CuO nanocomposite. The strong adhesion of this bonding layer improved the bonding degree of Al/CuO. Several groups have used PDA to modify or assemble energetic materials [20,21,22,23,24]. Zhu et al. have coated a shell of PDA on HMX particles in order to enhance the safety and adhesive properties of HMX when used in PBX [22]. He et al. synthesized n-Al/PDA/PTFE by PDA as an adhesive layer and the reactivity of n-Al@PDA/PTFE was regulated by adjusting the thickness of PDA [25]. While some successes have been reported, many of these techniques needed to be carried out under alkaline conditions (pH 8.5) [21,24,25,26,27]. However, Al NPs exhibit significantly higher chemical reactivity and can more readily react with water under alkaline conditions [28] In this paper, a novel, one-pot approach is proposed for the assembly of Al/PDA/CuO in a neutral aqueous solution at room temperature, which avoided energy loss due to the Al oxidation under alkaline conditions. Thermal analysis experiments indicated that the Al/PDA/CuO had enhanced heat release properties compared with physically mixed Al/CuO. As a bionic adhesive, polydopamine strongly adheres to metal and organic materials. Therefore, the effect of PDA on the assembly of HMX/PDA/Al/CuO and AP/PDA/Fe_2_O_3_ nanocomposites under mild conditions was further investigated, demonstrating that the thermal properties of these materials were significantly improved compared to their physically mixed counterparts. This new method is environmentally friendly and can be performed under mild reaction conditions. Therefore, this work offers a new research approach for the biological synthesis of other explosive materials and energetic nanomaterials.

## 2. Results and Discussion

### 2.1. Polydopamine Coated Nanoparticles

**Figure 1:**
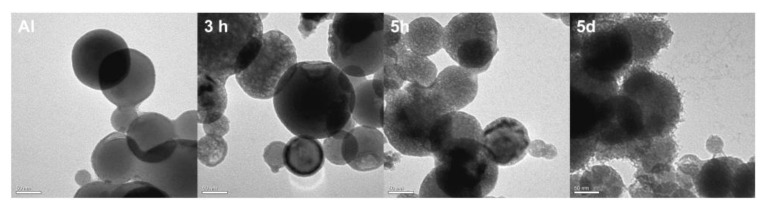
TEM micrographs of PDA-coated Al NPs after 0 h, 3 h, 5 h, and 5 d of polymerization in 0.01 M Tris-HCl at a pH of 8.5, respectively. Scale bars are 50 nm.

**Figure 2:**
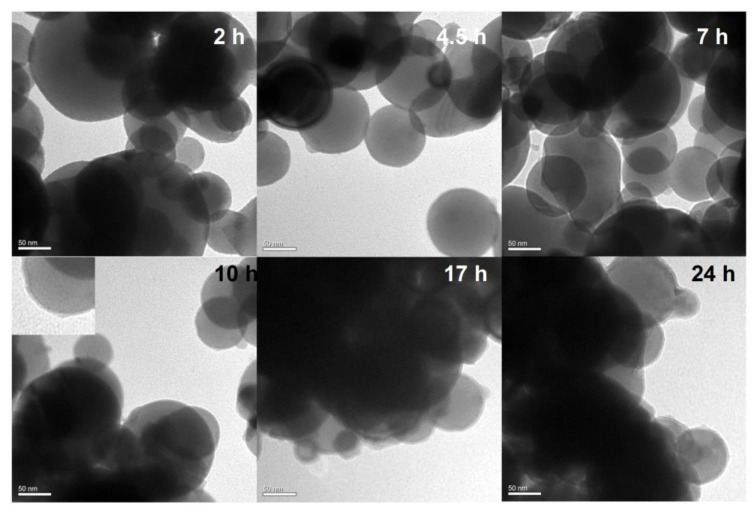
TEM micrographs of the PDA-coated Al NPs after 2 h, 4.5 h, 7 h, 10 h, 17 h, and 24 h of polymerization in 0.01 M PBS at a pH of 7.0, respectively. Scale bars are 50 nm.

**Figure 3:**
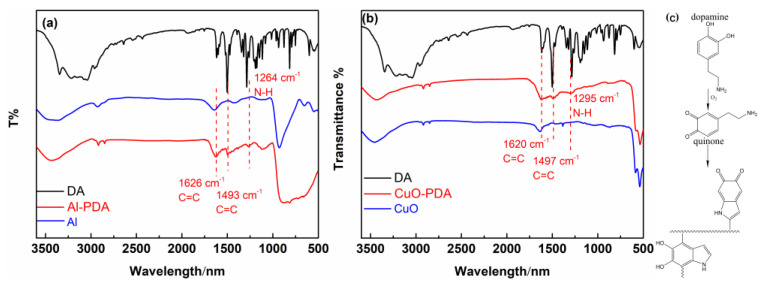
Transmission FTIR spectra of (**a**) Al, DA, and PDA-coated Al NPs, (**b**) CuO, DA, and PDA-coated CuO NPs, and (**c**) schematic structure of polydopamine.

Under alkaline conditions, DA can self-polymerize into PDA with strong adhesion on the surface of various materials [29]. However, Al NPs are extremely unstable in alkali environments due to the chemical activity of aluminum. The effect of the self-polymerization of DA on aluminum under alkaline conditions was evaluated by TEM (Figure 1). After a 3 h coating process, obvious corrosion damage was observed on the surface of the Al NPs. Therefore, coating Al NPs with PDA under alkaline conditions is not suitable. Therefore, we developed a novel approach for the formation of PDA on Al NPs in 0.01 M PBS at a neutral pH of 7.0. The Al NPs after this polydopamine coating process are shown in Figure 2. After 10 h of polymerization, the Al NP surface was coated with a thin polydopamine layer. After 24 h of polymerization, this layer had a thickness of 3.8 ± 0.5 nm. Therefore, PDA coating was successful under neutral conditions. Next, the FTIR analysis was used to investigate the DA and the Al NPs coated with PDA for 24 h in neutral conditions (Al-PDA-24 h) to verify that the surface coating consisted of PDA (Figure 3a). The FTIR spectrum of DA showed some strong absorption peaks at 1343 cm^−1^ (-CH_2_), 1320 cm^−1^ (C-O-H), 1190 cm^−1^ (C-O), and 1176 cm^−1^ (C-C). However, the FTIR spectrum of Al-PDA-24 h did not show these peaks. It was speculated that the DA underwent an intramolecular cyclization reaction and formed indole derivatives [30]. New absorption peaks were observed in the Al-PDA-24 h spectrum at 1626 cm^−1^ (C=C, aromatic ring absorption), 1493 cm^−1^ (C=C, aromatic ring absorption), and 1264 cm^−1^ (N-H groups of PDA) [29,31]. This demonstrated that the surface of the Al NPs was successfully coated with PDA.

**Figure 4:**
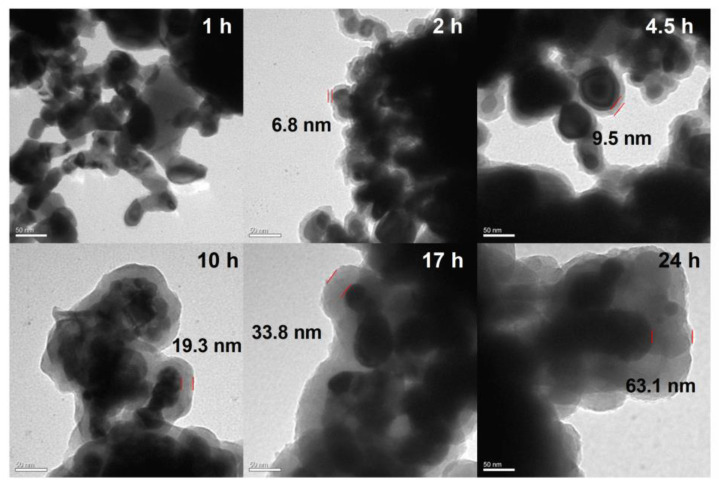
TEM micrographs of the PDA-coated CuO NPs after 2 h, 4.5 h, 7 h, 10 h, 17 h, and 24 h of polymerization in 0.01 M PBS at a pH of 7.0, respectively. Scale bars are 50 nm. Red line spacing represents PDA thickness.

CuO NPs were also coated in PDA under the same conditions (0.01 M PBS at a neutral pH of 7.0). The TEM images in Figure 4 demonstrate that these CuO NPs were well-coated in PDA. The thickness of the coating increased with increasing polymerization time. However, the deposition rate of PDA on the CuO NPs was much higher than that on the Al NPs. After 24 h of polymerization, the PDA coating on the CuO NPs had a thickness of 63.1 ± 2 nm. Next, the FTIR analysis was used to investigate DA and the CuO NPs coated with PDA for 24 h in neutral conditions. Compared with CuO and DA, new absorption peaks were observed in the CuO-PDA 24 h spectrum at 1620 cm^−1^ (C=C, aromatic ring absorption), 1497 cm^−1^ (C=C, aromatic ring absorption), and 1295 cm^−1^ (N-H groups of PDA) [29]. This demonstrated that the surface of the CuO NPs was also successfully coated with PDA.

### 2.2. Preparation of Al/PDA/CuO Composite

**Figure 5:**
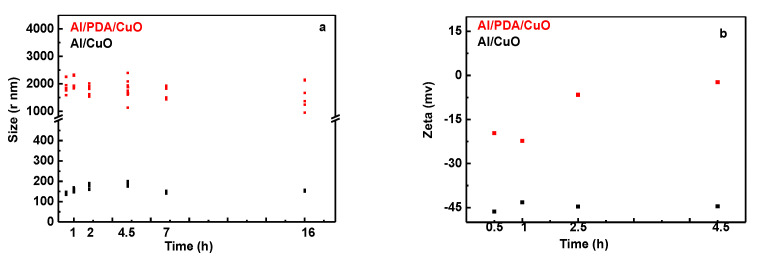
Influence of reaction time on hydrodynamic size (**a**) and zeta potential (**b**).

DA was added to the Al and CuO mixed solution and the polymerization of PDA was used as an intermediate transition layer to bring Al and CuO NPs into close proximity. The assembly of Al/PDA/CuO was evaluated by measuring the particle size (via DLS) and zeta potential with increasing reaction time, as shown in Figure 5 for different reaction times. Compared with Al/CuO prepared by physical mixing, the particle size of Al/PDA/CuO gradually increased with increasing DA coating time and the absolute value of the zeta potential gradually decreased. This indicated that the Al and CuO aggregated during the PDA assembly process, leading to an increase in the particle size. Therefore, the system presented an unstable state. This analysis shows that PDA promoted the assembly of Al and CuO in a dense structure. Because the growth of the carbon layer in Al/PDA/CuO reduced the energetic density and the NPs assembled by PDA self-polymerization tended to aggregate in 30 min, the best assembly time was determined to be 30 min.

**Figure 6:**
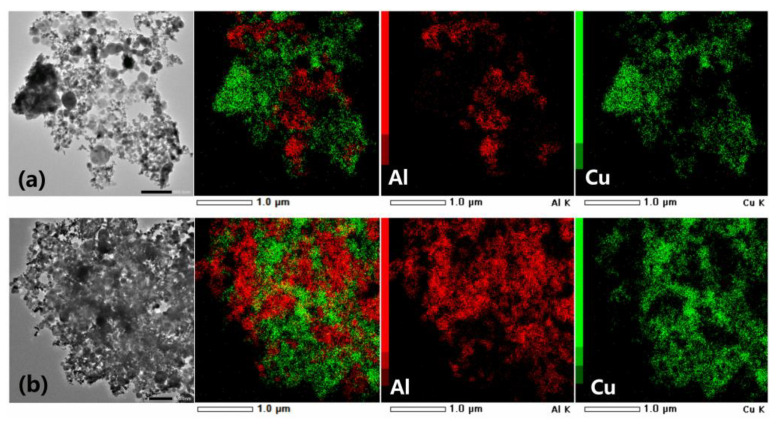
TEM micrographs of (**a**) physically mixed Al/CuO after 30 min and (**b**) Al/PDA/CuO after 30 min assembly time. Red represents Al element, green represents Cu element.

TEM images of Al/CuO and Al/PDA/CuO obtained at different magnifications are shown in Figure 6. The physically mixed CuO nanoparticles and Al nanoparticles were randomly distributed, and the same NPs tended to aggregate, as displayed in Figure 6a. In contrast, TEM images of Al/PDA/CuO showed CuO was closely integrated with Al, and both particles were evenly distributed (Figure 6b). This was potentially because the self-polymerization of PDA helped Al and CuO to bind together, enhancing the mixing uniformity.

### 2.3. Thermal Decomposition Performance of Al/PDA/CuO Energetic Nanocomposite

**Figure 7:**
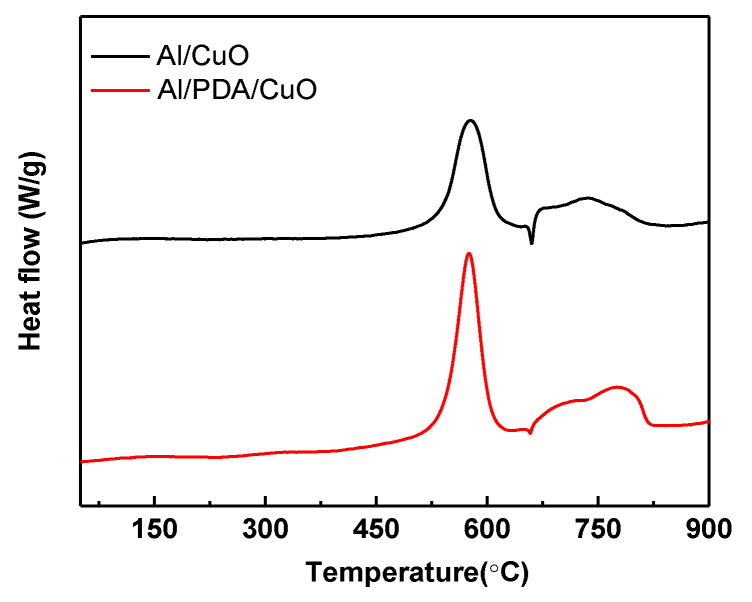
DSC curves of physically mixed Al/CuO after 30 min and Al/PDA/CuO after 30 min assembly time.

Simultaneous TGA and DSC were performed under nitrogen to compare the energetic properties and energy output efficiency of physically mixed Al/CuO and the Al/PDA/CuO composite prepared via PDA assembly, as shown in Figure 7. The obtained DSC curves showed exothermic peaks between 400 and 827 °C. These peaks were split into two sections due to the endothermic peak of Al at 658 °C. In the Al/PDA/CuO curve, a sharp exothermic peak (1398.2 J/g) was observed before the endothermic Al melting peak. This peak was attributed to a solid–solid reaction. After the Al melting peak, one broad exothermic peak attributed to a solid–liquid reaction was observed (671.5 J/g). Therefore, the total reaction heat of Al/PDA/CuO was about 2069.7 J/g. In contrast, the heat release from the physically mixed Al/CuO was only 1438.9 J/g. This result reveals that the PDA binding layer significantly enhanced energy output by aiding the formation of a highly uniform morphology with a reduced mass–transport distance.

### 2.4. PDA-Assembled Inorganic Oxidant/Nanometal Catalyst AP/Fe_2_O_3_

**Figure 8:**
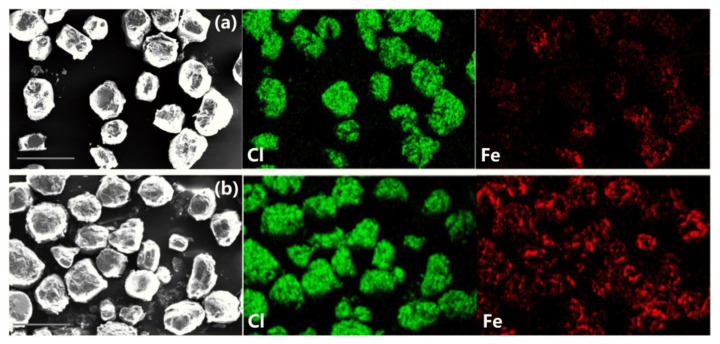
SEM and EDS mapping images of (**a**) AP/Fe_2_O_3_ and (**b**) AP/PDA/Fe_2_O_3_. Scale bars are 500 μm. Red represents Fe element, green represents Cl element.

To illustrate the generality of the PDA self-polymerization approach, PDA was used to assemble AP/PDA/Fe_2_O_3_. SEM images of the prepared AP/PDA/Fe_2_O_3_ and physically mixed AP/Fe_2_O_3_ are shown in Figure 8a,b. Compared with physically mixed AP/Fe_2_O_3_, more Fe_2_O_3_ was distributed on the surface of AP (AP could be determined by the distribution of Cl element). Therefore, the use of PDA promoted the formation of a homogeneous complex between AP and Fe_2_O_3_.

**Figure 9:**
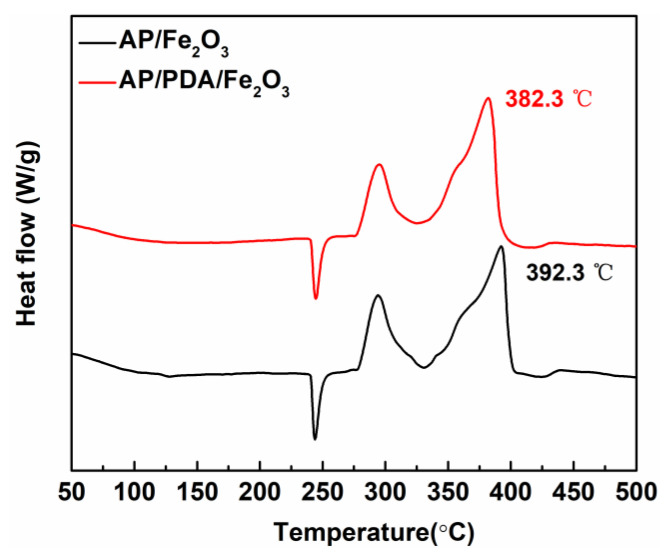
DSC curves of AP/Fe_2_O_3_ and AP/PDA/Fe_2_O_3_.

The strong adhesion properties of PDA promoted the assembly of AP and Fe_2_O_3_. To study the influence of the PDA assembly process on catalytic activity, the thermal properties of AP/PDA/Fe_2_O_3_ and physically mixed AP/Fe_2_O_3_ samples were evaluated by DSC, as shown in Figure 9. Three peaks were observed for the thermal decomposition of AP/PDA/Fe_2_O_3_. The orthorhombic to cubic phase transition of AP was responsible for the endothermic peak observed at 244.7 °C. The exothermic peak at 294.9 °C was ascribed to the low-temperature decomposition (LTD) of AP. The exothermic peak at 382.3 °C was ascribed to the high-temperature decomposition (HTD) of AP. Compared with physically mixed AP/Fe_2_O_3_, the HTD peak of AP/PDA/Fe_2_O_3_ was shifted to a lower temperature by 10.0 °C. Therefore, in addition to promoting the formation of a homogeneous AP and Fe_2_O_3_ composite, the use of PDA significantly enhanced the catalytic effect of Fe_2_O_3_ on AP.

### 2.5. PDA-Assembled Organic Energetic Material: HMX/Al/CuO Nanothermite

**Figure 10:**
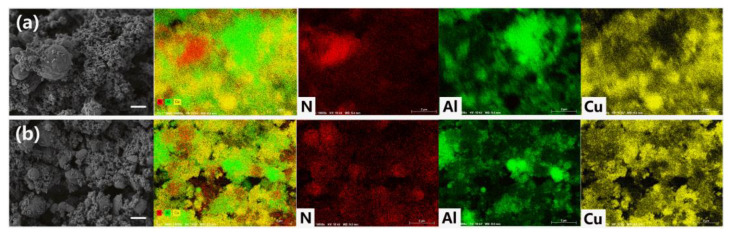
SEM micrographs and EDS mapping of (**a**) physically mixed HMX/Al/CuO and (**b**) HMX/PDA/Al/CuO. Scale bars are 1 μm. Red represents N element, green represents Al element, yellow represents Cu element.

The PDA assembly process was also used to prepare HMX/PDA/Al/CuO in a neutral aqueous solution. As shown in Figure 10, achieving close contact between HMX, Al, and CuO is crucial for preparing HMX/Al/CuO energetic nanocomposites with good performance. The morphologies of HMX/Al/CuO and HMX/PDA/Al/CuO were evaluated by SEM, as shown in Figure 10. Physically mixed HMX/Al/CuO exhibited poor inter-component homogeneity and significant agglomeration of each single component. In contrast, the three components of HMX/PDA/Al/CuO were observed to overlap to a high degree. EDS analysis showed that N, Cu, and Al were uniformly distributed, indicating the beneficial influence of PDA during the assembly of this energetic nanocomposite.

**Figure 11:**
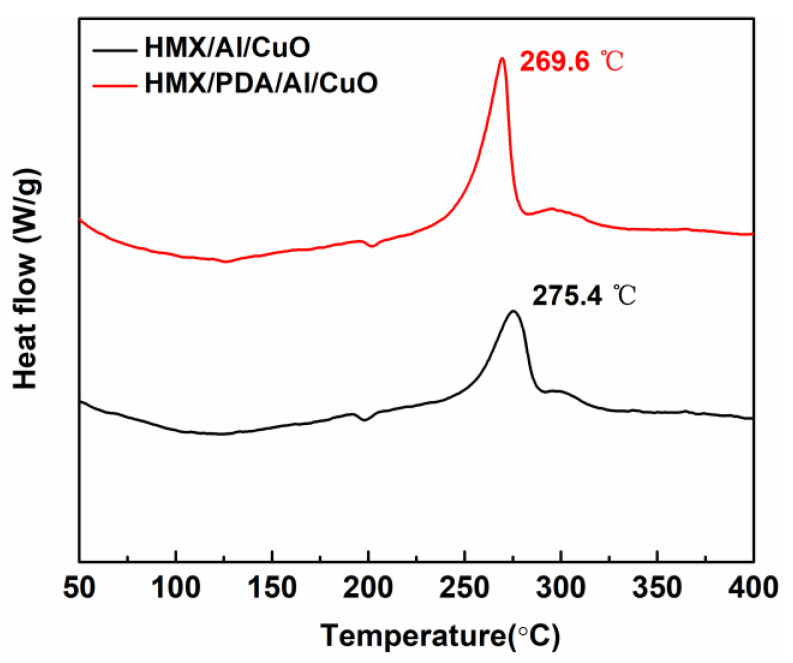
DSC curves for HMX/Al/CuO and HMX/PDA/Al/CuO.

To further study the catalytic behavior of the catalysts for HMX, the DSC curves of HMX/Al/CuO and HMX/PDA/Al/CuO were obtained (Figure 11). The exothermic peak of HMX/PDA/Al/CuO at 269.6 °C was attributed to the decomposition of HMX, and this peak was 5.8 °C lower than that of HMX/Al/CuO. These results indicate that the reactivity of HMX was enhanced by utilizing PDA to assemble this nanocomposite.

In previous studies, we investigated an energetic composite denoted HMX@Al@CuO, which was prepared by coating HMX with a PDA layer followed by grafting the new peptide SH-25-NH_2_ onto the PDA-functionalized HMX [32]. Based on the obtained results, we believe that in addition to assembling energy-containing materials by adhesion under neutral conditions, PDA could also be used to modify biomolecules such as proteins, DNA, and peptides. This would realize the orderly and tight assembly of nanocomposites with the help of biomolecular targeting.

## 3. Materials and Methods

### 3.1. Materials

CuO NPs (20 nm) and Fe_2_O_3_ NPs (50 nm) were obtained from Shanghai Yunfu Nanotechnology Co., Ltd. (Shanghai, China). Al NPs (100 nm, 4.3 ± 0.3 nm thin alumina surface film) were provided by Nano Material Engineering Company (Jiaozuo, China). HMX and AP were synthesized in our laboratory. 3-Hydroxytyramine hydrochloride (DA-HCl) was provided by Aladdin Reagent Co., Ltd. (Shanghai, China). Tris–HCl (0.1 M, pH 8.5) and phosphate buffer (0.1 M, pH 7.0) (PBS) were supplied by Beijing Solarbio Science & Technology Co., Ltd. (Beijing, China).

### 3.2. Methods

Preparation of the dopamine-modified Al NPs (or HMX) under alkaline conditions: 32 mg of Al NPs were added to 4 mL ultrapure water and ultrasonicated for 5 min (2 s pulses with 1 s gaps, 100 W). After ultrasonication, this mixture was added to 4 mL of dopamine–Tris HCl (4 mg mL^−1^, pH 8.5, 20 mM Tris buffer). This mixture was incubated for a certain length of time at room temperature.

Preparation of the dopamine-modified Al NPs (or CuO NPs or HMX) under neutral conditions: 20 mg of Al NPs or CuO NPs was added to 5 mL of PBS solution (0.01 M, pH 7.0) and ultrasonicated for 5 min (2 s pulses with 1 s gaps, 100 W). This was followed by the addition of 10 mg of DA. The reaction was allowed to proceed at room temperature.

Preparation of Al/PDA/CuO: 20 mg of Al NPs and 20 mg of CuO NPs were each separately added to 5 mL of PBS solution (0.01 M, pH 7.0). These mixtures were ultrasonicated for 5 min (2 s pulses with 1 s gaps, 100 W) to achieve homogenous dispersions. Next, the dispersed Al and CuO solutions were combined (33:92 volume ratio). DA (2 mg/mL) was then added to the Al/CuO mixed solution. This mixture was stirred for a certain length of time at room temperature. Finally, the Al/PDA/CuO was obtained after centrifugation (10 min, 10,000 rpm) and washing with ultrapure water three times. For comparison, a physically mixed Al/CuO nanoenergetic sample was prepared without the addition of DA. These samples were dried at 30 °C for 2 d.

Preparation of HMX/PDA/Al/CuO: 20 mg of Al NPs, CuO NPs, and HMX were each separately added to 5 mL of PBS solution (0.01 M, pH 7.0). These mixtures were ultrasonicated for 5 min (2 s pulses with 1 s gaps, 100 W) to achieve homogenous dispersions. Then, the Al, CuO, and HMX dispersions were mixed in a volume ratio of 33:92:125, and DA (2 mg/mL) was added to this mixed solution. The as-obtained mixture was stirred for 30 min at room temperature. Finally, HMX/PDA/Al/CuO was obtained via centrifugation (10 min, 10,000 rpm). For comparison, a physically mixed HMX/Al/CuO nanoenergetic sample was prepared without the addition of DA. These samples were dried at 30 °C for 2 d.

Preparation of AP/PDA/Fe_2_O_3_: 30 mg of Fe_2_O_3_ NPs was added to a saturated solution consisting of 3 mL PBS solution (0.01 M, pH 7.0) and an appropriate amount of AP. This mixture was ultrasonicated for 5 min (2 s pulses with 1 s gaps, 100 W). Then, 2.1 mL of a saturated AP solution containing 30 mg DA and 300 mg AP was added to the 0.9 mL Fe_2_O_3_ solution. The as-obtained mixture was stirred at room temperature for 60 min. Finally, the AP/PDA/Fe_2_O_3_ was obtained via centrifugation (10 min, 10,000 rpm). For comparison, a physically mixed AP/Fe_2_O_3_ nanoenergetic sample was prepared without the addition of DA. These samples were dried at 30 °C for 2 d.

### 3.3. Characterization

DLS and zeta potential (ζ) test: DLS and ζ test were performed with a Malvern Zetasizer NanoZS90 instrument (Nano ZS90, Malvern Panalytical, Malvern, UK).

Morphological analysis: The morphological features of the nanoenergetics were evaluated by high-resolution TEM (JEOL-2100F) and SEM (S-4800, Hitachi, Tokyo, Japan). Both electron microscopes were coupled with energy-dispersive X-ray spectrometers.

Surface chemical analysis: The samples were evaluated by FTIR (Nicolet 6700 FTIR Spectrometer, Thermo Fisher, Waltham, MA, USA).

Thermal analysis: The thermal properties of the samples were evaluated by DSC (TGA/DSC 3+, Mettler Toledo, Greifensee, Switzerland). Heating rates of 10 °C·min^−1^ were used to obtain DSC curves in the range of room temperature to 900 °C under a N_2_ atmosphere.

## 4. Conclusions

In summary, PDA was utilized for the room-temperature self-assembly of CuO NPs and Al NPs in an aqueous phosphate buffer under neutral conditions. This method avoided the aluminum oxidation problem caused by traditional DA self-polymerization under alkaline conditions. The adherence properties of PDA improved interfacial contact between the Al NPs and CuO NPs. Consequently, the prepared Al/PDA/CuO energetic nanocomposite exhibited a heat release of 2069.7 J/g, which was 1.44 times higher than the heat release from physically mixed Al/CuO. To further investigate the versatility of polydopamine for assembling different types of energetic nanocomposites. HMX/PDA/Al/CuO and AP/PDA/Fe_2_O_3_ energetic nanocomposite materials were also prepared by PDA self-assembly. The use of PDA enhanced the peak thermal decomposition temperatures of both HMX and AP. In summary, using polydopamine to encapsulate energetic materials under mild and neutral preparation conditions is a versatile and facile method for the introduction of various functionalities. This preparation method shows excellent promise for the simple and green assembly of energetic materials with or without Al NPs under mild reaction conditions.

## Data Availability

The data presented in this study are available on request from the corresponding author.
